# Reserves in Context: Planning for Leakage from Protected Areas

**DOI:** 10.1371/journal.pone.0129441

**Published:** 2015-06-08

**Authors:** Anna R. Renwick, Michael Bode, Oscar Venter

**Affiliations:** 1 ARC Centre of Excellence for Environmental Decisions, the NERP Environmental Decisions Hub, Centre, for Biodiversity & Conservation Science, University of Queensland, Brisbane, Queensland, Australia; 2 ARC Centre of Excellence for Environmental Decisions, School of Botany, University of Melbourne, Melbourne, Australia; 3 Centre for Tropical Environmental and Sustainability Science and the School of Marine and Tropical Biology, James Cook University, Cairns, Australia; Potsdam Institute for Climate Impact Research, GERMANY

## Abstract

When protected areas reduce threats within their boundaries, they often displace a portion of these threats into adjacent areas through a process known as ‘leakage’, undermining conservation objectives. Using theoretical models and a case study of terrestrial mammals in Indonesia, we develop the first theoretical explanation of how leakage impacts conservation actions, and highlight conservation strategies that mitigate these impacts. Although leakage is a socio-economic process, we demonstrate that its negative impacts are also affected by the distribution of species, with leakage having larger impacts in landscapes with homogeneous distribution of species richness. Moreover, leakage has a greater negative effect when conservation strategies are implemented opportunistically, even creating the potential for perversely negative consequences from protected area establishment. Leakage thereby increases the relative benefits of systematic conservation planning over opportunism, especially in areas with high leakage and heterogeneously distributed species. Although leakage has the potential to undermine conservation actions, conservation planning can minimize this risk.

## Introduction

The human alteration of natural landscapes is driving a global biodiversity crisis [1]. Nature reserves, which serve to protect biodiversity features from the processes that threaten them, have become the cornerstone of biodiversity conservation [2]. When properly managed, reserves can effectively reduce habitat loss and degradation within their bounds [3, 4], but the ability of reserves to reduce net biodiversity loss depends on more than internal management actions [5]. The conservation benefits of reserves also depend on changes that occur in the surrounding landscape. Reserves can lead to negative consequences for conservation outcomes beyond the reserve borders. For example, conservation purchases can raise the value of adjacent lands, thereby reducing the cost-effectiveness of future conservation purchases [6]. Human demand for ecosystem goods and services is the primary cause of habitat degradation [7]. Reserves reduce the supply of these goods and services, but do not address the underlying demand, which is instead displaced, partly or completely, into other areas [5, 8–11]. The increase in threatening processes on unprotected land following the creation of a reserve is known as ‘leakage’ [5].

Leakage can and does occur at a range of scales, from local to international. If reserves are established adjacent to lands that have high biodiversity value, leakage of localized threats into these areas can negatively affect biodiversity outcomes [6, 12]. At larger scales, countries that protect their national assets, for example by preventing deforestation, may cause displacement of the driver of change, for example logging, into other countries [13]. Recent studies have attempted to quantify leakage at both local and national scales. Oliveira et al. [8] showed that while forest reserves lead to reduced rates of deforestation within their borders, deforestation in adjacent unrestricted land increased by 300–470%, much of which could be attributed to uncontrolled clear-cutting around the new reserve areas. Meyfroidt and Lambin [13] found that almost 40% of forest regrowth that occurred in Vietnam as a result of logging restrictions in the country was offset by increased extraction in other countries supplying timber to the Vietnamese market. Similarly, Gan and McCarl [14] showed that when logging is reduced in one country, between 42–95% of this reduction may be leaked as increased logging into other countries. Leakage is also evident in the marine environment as marine protected areas often result in displaced fishing effort [15–17]. The scale of this leakage points to an influential conservation process.

Leakage processes are well-known in other environmental management contexts, such as carbon offsetting (e.g., decommissioning coal fired power plants or protecting high carbon forests). In carbon offsetting, the negative impacts of leakage on emissions reductions have been long recognized [18], and tools to monitor leakage impacts on overall emissions reductions have been developed [19]. However, these tools are not explicitly intended to limit the amount of leakage, but instead provide an *ex-post* estimation of the impacts of leakage on net carbon emissions, and lead to a proportional reduction in the carbon credits awarded. While lessons can be drawn from these tools, they do not explain how leakage can be incorporated into conservation decision making.

Conservation scientists have developed techniques to prioritize areas for reserve establishment [2, 20], broadly labeled systematic conservation planning. These approaches rank or select areas based on the biodiversity features they contain, the threats they face, and increasingly, the costs involved in securing their protection [2, 21]. Within the field of conservation biology, leakage needs to be better understood to prevent it negating the positive benefits of conservation action. Here we use a modeled conservation landscape to investigate the dynamics and impacts of conservation leakage. A modeled landscape allows us to explore how different levels of leakage intensity and different spatial patterns of species richness affect the benefits anticipated from systematic conservation planning. Conservation also involves opportunistic additions to existing reserve networks, and we therefore test the efficiency of incorporating leakage into opportunistically expanding reserve networks. We further illustrate and explore each of these questions using a case study from Kalimantan, Indonesia.

## Methods

### 2.1 The conservation landscape

We create a grid of *P* land parcels, with each parcel in the landscape characterized by two primary factors. The first is the local species richness of each parcel *i*, measured by an amount *S*_*i*_ that is distributed normally with mean species richness *m* and standard deviation *s*. The second defining feature is the threat faced by each parcel, measured by the probability of habitat conversion, *q*_*i*_, over a finite interval (e.g., 10 years). In the absence of conservation actions, and with spatially homogeneous threats (i.e., *q*_*i*_ *= q*), the expected number of converted habitat parcels over this interval is therefore *qP*.

### 2.2. Leakage and reserve system design

We first model and evaluate leakage within a systematically planned reserve network. A single conservation organization is able to acquire land sufficient to create *R* reserves in a once-off set of acquisitions. We assume that the cost of land acquisition is spatially homogeneous, and therefore planners will choose to protect the *R* land parcels that have the highest species richness.

We assume that reserves are sufficiently large, and management is sufficiently effective that no species are lost from within protected parcels. In the absence of any leakage, the parcels that remain unprotected would be exposed to the original probability of loss (*q*), and we would therefore expect to see the degradation of *q*(*P*—*R*) parcels. Conservation actions would therefore have averted the loss of *qR* parcels. In the presence of leakage, some proportion of this averted loss would be displaced into unprotected parcels in the landscape. We define leakage as λ, the proportion of habitat loss that was going to happen on protected areas, that instead is displaced outside the protected areas. This displaced threat is shared equally by all parcels that remain unprotected. An amount of leakage λ will therefore effectively increase the probability of loss for unprotected parcels, such that the total expected number of lost parcels is the sum of the degradation that was already going to occur outside the reserves, added to the additional leaked degradation: *q*(*P*—*R*) + λ *qR*. Given that there are only *P-R* parcels that can still be degraded, the new probability of loss (q_L_) for each unprotected parcel is therefore calculated as:

$$q(P-R)+q\lambda R=q_{L}(P-R)$$ (1a)

and therefore:

$$q_{L}=q\left(1+\frac{\lambda R}{P-R}\right)$$ (1b)

The expected amount of habitat loss in the presence of leakage, *D*_*L*_, is therefore a function of the protected area network size, the original threat rate, and the leakage rate:

$$D_{L}=q_{L}\left(P-R\right)=q\left(1+\frac{\lambda R}{P-R}\right)\left(P-R\right)=q\left(P-R\left(1-\lambda \right)\right)$$ (2)

where degradation is modeled as a random binomial process across the unprotected landscape. With a known landscape distribution of species *S*_*i*_, Eq (2) allows us to calculate the expected amount of species that are (a) protected, (b) unprotected and extant, and (c) degraded. The number of protected species is equal to a weighted integral of the upper tail of the species distribution *S*_*i*_, since managers will preferentially choose the most species rich parcels to protect. The threshold that defines the upper tail of the species distribution is chosen using an iterative search, since there is no closed formula for the inverse of the cumulative distribution of a normal distribution [22]. The total remaining species are split between degraded and unprotected in proportions determined by the new loss probability *q*_*L*_.

We simulate the outcome of conservation planning in this model across three dimensions of variation. First, we simulate outcomes under a wide range of protection budgets, from 0% to 50% of the landscape. Second, we model three levels of leakage intensity: where leakage is absent (λ = 0), where leakage is 50% of all protection (λ = 0.5), and where leakage is total (λ = 1). Finally, we consider two different conservation landscapes with different levels of species heterogeneity—one with high parcel-to-parcel variation in species richness (*s =* 30), and the other where most parcels contain similar amounts of species richness (*s =* 5). In both landscapes the average amount of species in a land parcel is the same (*m =* 80). In each case we calculate the proportion of the original species richness that we would expect to remain extant within the landscape, after managers have created their desired protected areas and degradation (including the effects of leakage) has occurred outside the protected area network.

### 2.3. Leakage and opportunistic protection

Although conservation planning can involve the identification and construction of optimal reserve networks, in many situations conservation planners may only be able to purchase individual parcels in an opportunistic manner, as they become available on the land market. We use our landscape model to consider how leakage will affect the additional benefit of opportunistically adding land parcels to the conservation portfolio. Planners are offered the opportunity to purchase a single parcel of land with known species richness value *So*, and which has a probability *q* of being degraded. In the absence of any protection, this landscape would expect to lose an amount of species equal to:

$$L_{u}=qmP$$ (3)

where *m* is the species richness of the average parcel. With protection, and in the presence of leakage, the expected loss of species richness would become:

$$L_{p}=q\left(1+\lambda /\left(P-1\right)\right)\left(mP/\left(P-1\right)-S_{O}/\left(P-1\right)\right)\left(P-1\right)$$ (4)

The first term in this equation, *q* (1 + λ / (*P - *1)), is equal to Eq (1) when the reserve network contains only one parcel, and represents the new probability of loss for unprotected parcels given the leakage from the newly protected parcel. The second term, (*mP* / (*P - *1)—*S*_*o*_ / (*P - *1)), gives the average species richness of the unprotected parcels, now that the parcel with species richness *S*_*o*_ has been protected. The final term represents the number of parcels that remain vulnerable to degradation in the new system. The product of these three terms is therefore directly analogous to Eq (3). The resulting change in species richness could be either negative or positive, since unprotected areas now have a higher probability of loss as a result of leakage as shown in Eq 1, but this is spread across fewer unprotected parcels, and these may have a higher or lower average species richness, depending on the species richness of the protected parcel.

Planners aim to create reserves that will reduce biodiversity loss. For this to occur, land should only be purchased when the parcel’s species richness exceeds a critical threshold, which is a function of the leakage rate:

$$S_{0}>mP\left(1-\frac{P-1}{P-1+\lambda }\right)$$ (5)

For example, Eq (5) states that in a landscape of 100 land parcels, with a leakage rate of 10%, managers should only purchase parcels above the 11^th^ percentile in species richness otherwise conservation effort could theoretically cause greater landscape-wide loss of species.

### 2.4. Application of methods to Kalimantan

To illustrate our models in a real landscape, we repeat our analyses of leakage using a case study of Kalimantan, Indonesia (4.39°N, 108.83°E and 4.17°S, 118.99°E). Kalimantan is divided into *P* = 5697 grid cells of 10 km x 10 km. These grid cells operate as our land parcels, and represent the fundamental resolution of both protection and degradation. We therefore assume that these two processes operate homogeneously within parcels (i.e., degradation and protection affect the entirety of a given parcel). Land parcels could alternatively be described by cadastral boundaries (i.e., existing properties), given that these are likely to be the scale at which protection or degradation occurs.

We denote the presence of species *j* in land parcel *i*, by setting *S*_*ij*_ = 1. The *S*_*ij*_ values are based on distribution data of all restricted range mammals in Indonesia [23]. We choose to define ‘restricted range’ as all mammals that occur over less than 30% of Indonesia’s land area [24]. This threshold was arbitrarily chosen, and higher thresholds are likely to lead to an inclusion of more species and result in lower rates of modeled species loss. However, these factors are unlikely to alter our findings qualitatively. Overlaying the range maps for all 658 native land mammals in Indonesia, we find 567 of these to be restricted range, and these mammals were included for analyses. For each restricted range mammal, we used its range map to determine if it was present or absent within each parcel. In the absence of reservation, each land parcel is assumed to have an equal probability *q* of habitat conversion.

We consider the potential conservation outcomes that can be achieved by a single conservation actor creating a reserve network in the region. The planner aims, in the presence of leakage, to choose a set of protected areas ***R*** that maximizes extant species richness (i.e., undegraded land, both protected and unprotected):

$$\underset{R}{\text{max}}{\sum_{j=1}^{S}{\left[\sum_{i\in R}S_{ij}+(1-q_{L})\sum_{i\notin R}^{}Sij\right]}^{Z}}^{}$$ (6)

In this function, each species (identified by the subscript *j*) contributes independently to the objective. The term in the square parentheses represents the expected number of times species *j* will occur in the protected landscape. The first summation is the number of times *j* occurs in protected areas, and the second summation is the number of times we expect it to occur outside protected areas (i.e., given that it will be lost through degradation from a proportion of those parcels). The exponent 0> *z >*1 is used to ensure that the set of protected areas is representative—that it contains as many different species as possible. It means that the benefits achieved through increasing protection deliver diminishing marginal returns. Basically, it ensures that a manager choosing new reserves will preference the protection of species that have the least representation in the existing reserve network [25]. The total size of the protected area network, *P*, is equal to the conservation budget since all parcels are assumed to have equal, unit cost.

We apply this objective to a range of areas of protected land, for three different leakage rates. Unlike the previous models where we described the species richness in a landscape with continuous distributions, there are a finite number of land parcels in Kalimantan with known specie richness values. As a result, species richness outcomes vary, depending on the particular land parcels that are cleared. Once managers create a protected area network, the parcels that remain unprotected are subjected to loss with probability *q*_*L*_. We repeat this loss process 10,000 times, and plot both the average outcome and 95% confidence intervals around it.

## Results

### 3.1. Leakage and reserve system design

**Fig 1:**
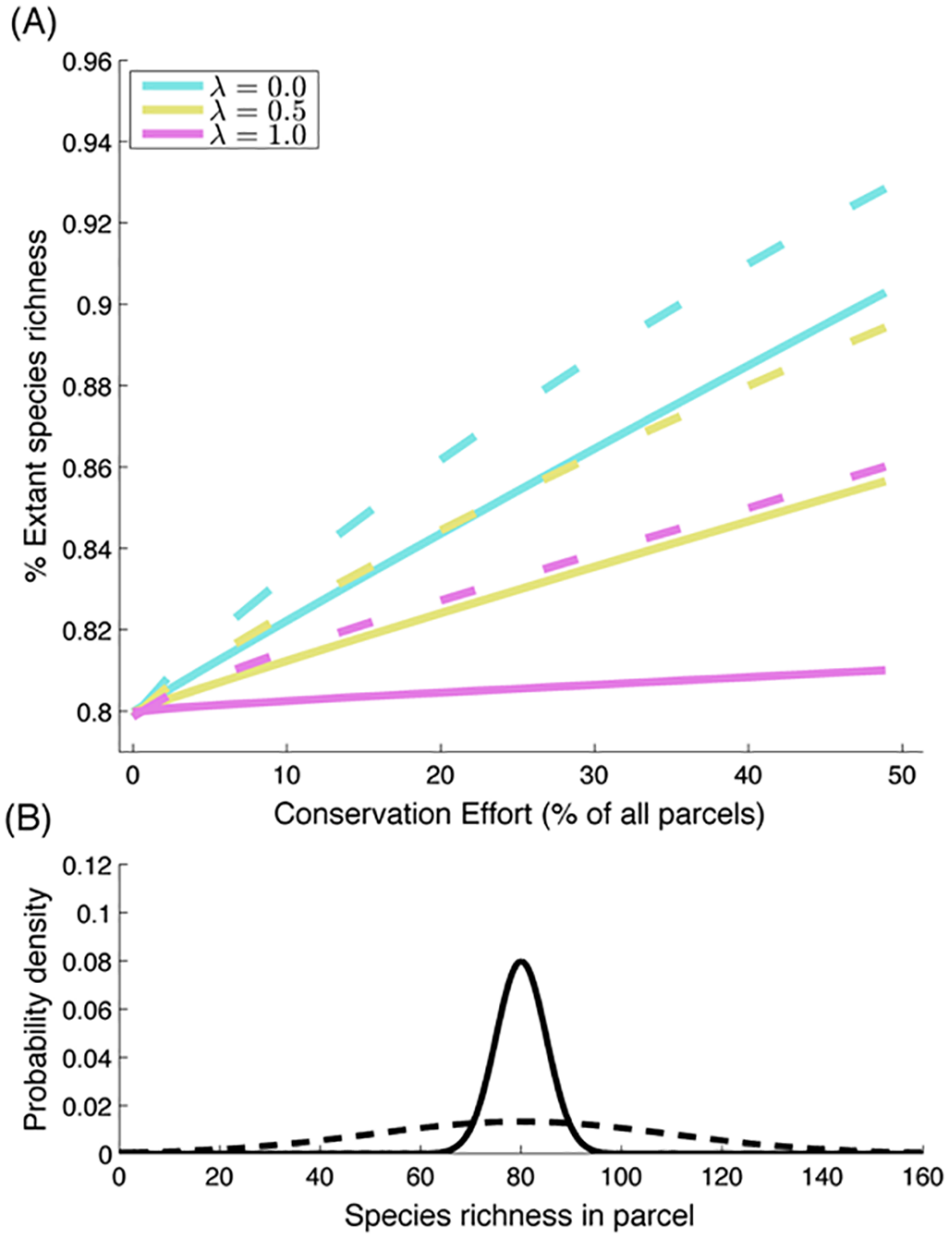
(A) Species richness conserved with increasing area of land conserved for different levels of leakage within heterogeneous (solid lines) and homogeneous (dotted lines) landscapes. (B) Results are for landscapes with highly variable (solid line, where s = 30) and relatively homogeneous (dotted line, where s = 5) distributions of species. The probability density function describes the probability (y-axis) that the species richness in a randomly chosen parcel will be any of the values on the x-axis. This figure is based on a landscape with ***P* = 225** parcels, with average species richness ***m* = 80**, and a background threat rate of ***q* = 0.2**.

In a systematically planned reserve network, the species richness conserved through the establishment of new reserves decreases as leakage rates increase (Fig 1A). Leakage undermines conservation outcomes by increasing the degradation rate of unprotected parcels. The distribution of species across a landscape (heterogeneous or more homogenous—Fig 1B) alters the effect of leakage. The negative effects of leakage are greatest in landscapes where the distribution of species is more homogenous (Fig 1A). Additional funding results in a diminishing but positive marginal change in extant species richness.

### 3.2. Leakage and opportunistic protection

**Fig 2:**
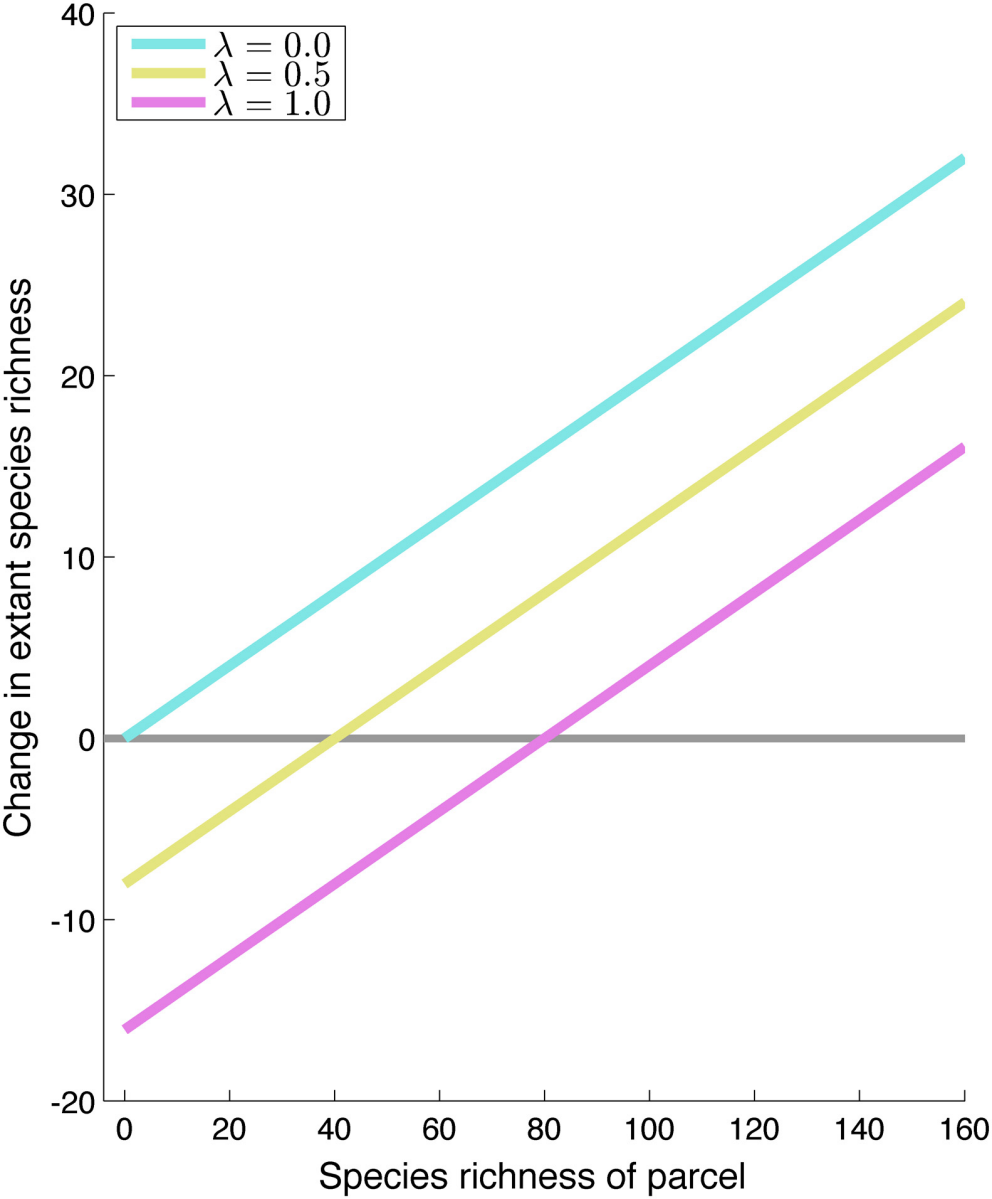
Species loss that would be averted by protecting a single parcel of land with known species richness (*S*_*0*_, on the x-axis). Results are shown for three different levels of leakage. Protecting parcels with relatively low species richness can result in a net loss of species richness. This figure is based on a landscape with ***P* = 225** parcels, with average species richness ***m* = 80**, and a background threat rate of ***q* = 0.2**.

When single additional reserves are added to an existing network of protected areas, the net biodiversity benefit is dependent on the species richness of the new area relative to the average species richnes in unprotected parcels, and the leakage rate (Fig 2). Purchasing an opportunistically available parcel of land is always beneficial if leakage is not present (λ = 0). However, opportunistic acquisition has a perverse net negative effect on extant species richness if the site’s species richness is sufficiently lower than the landscape average.

The decision over whether to purchase land for biodiversity is dependent on the biodiversity of the average unprotected parcel in the landscape (*m*): in the presence of leakage, protection is only beneficial for parcels with relatively high species richness, since leakage will transfer some of the threat onto the remaining unprotected parcels. Above the threshold given in Eq (6), protection results in a net positive averted loss: *A = ΔB*_*u*_-*ΔB*_*p*_ >0. While the level of threat, *q*, does not influence the threshold in Eq 6, it does influence the magnitude of impacts following protection. In the case where there is no threat, the positive or negative impacts of a protected area are negated, and as threat increases, these impacts become amplified.

### 3.3. Application of methods to Kalimantan

**Fig 3:**
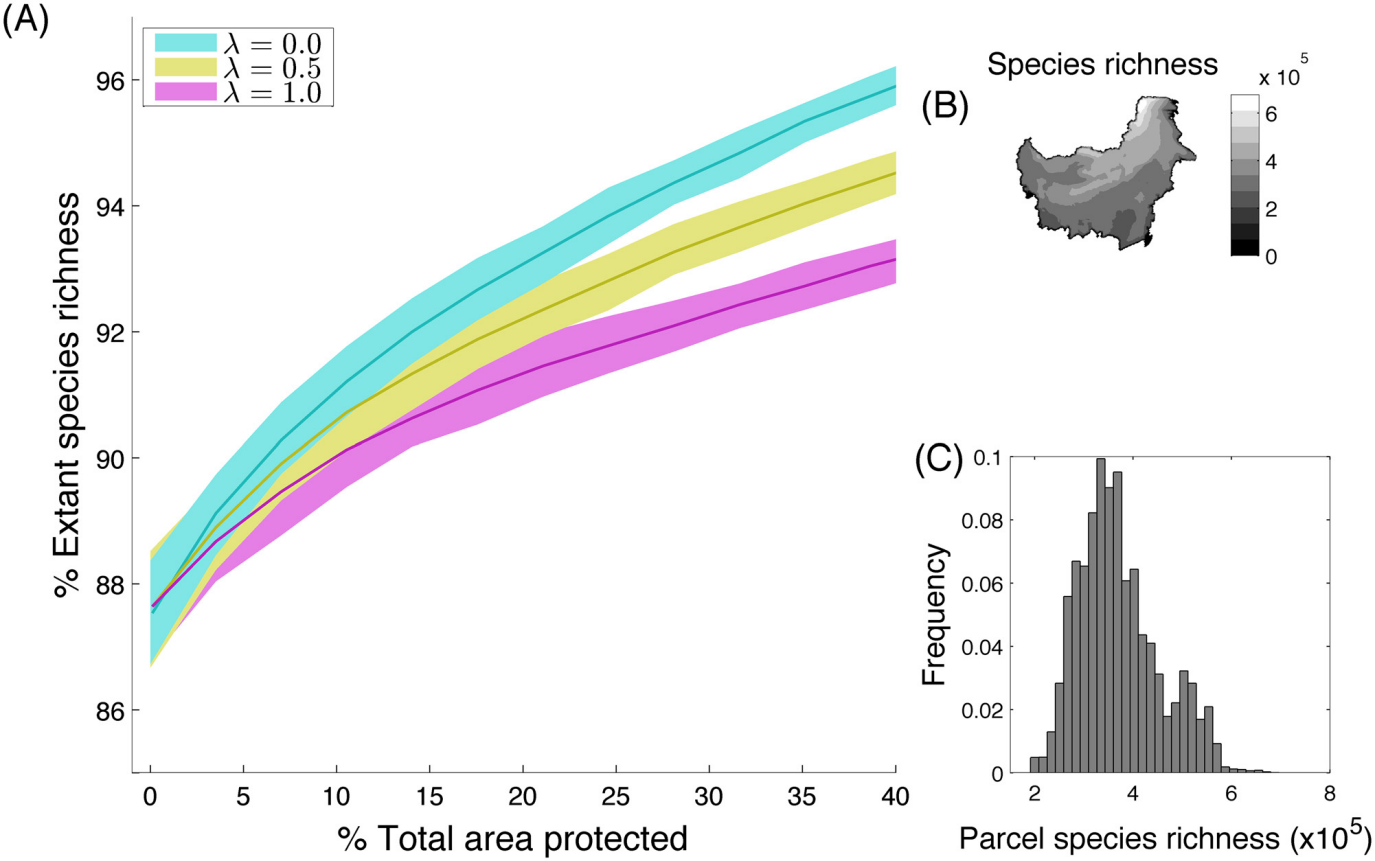
(A) Biodiversity objective under increasing area protected, for different levels of leakage (markers). This figure is based on the distribution of restricted range mammals in Kalimantan, Indonesia and a background threat rate of ***q* = 0.2** and ***z* = 0.75**. Solid lines indicate the average outcome of 10,000 stochastic land parcel loss simulations, and the shaded regions encompass 95% of the realisations. (B) Spatial distribution of species across 5697 land parcels in Kalimantan. (C) Relative frequency distribution of local species richness in Kalimantan land parcels.

The Kalimantan case study showed similar patterns to the simulated landscapes, both in the inputs (spatially variable species richness; Fig 3B and 3C), and the consequences of leakage. With an increasing area conserved, a greater number of species can be protected (Fig 3A), but leakage reduces the magnitude of this benefit (Fig 1). Systematic protection always results in a net benefit to species richness (even when λ = 1), since leakage displaces threats from the most species rich parcels onto parcels with lower species richness.

## Discussion

Our results demonstrate theoretically how much, and in what circumstances, leakage can undermine biodiversity conservation outcomes on a landscape scale. Even under complete leakage, net conservation benefits can still be achieved by the systematic protection of areas with the highest species richness, as the displacement of degrading activity will flow into areas with lower species richness. In landscapes with a heterogeneous distribution of species, this preferential choice of high species richness parcels results in a higher net gain from conservation actions than that in a more homogenous environment, where the degrading activities are displaced onto land with similar species richness values. In the presence of complete leakage (λ = 1), the establishment of a protected area in a completely homogenous landscape (i.e., where species richness *S*_*i*_ has a standard deviation of *s* = 0) would make no difference—positive or negative—to the total amount of extant species richness. Heterogeneity in the patterns of species richness therefore plays a crucial role in determining the impacts of leakage, and its importance increases as leakage increases. This result was apparent in both the simulated landscapes and in Kalimantan, Indonesia. The benefits of protecting an area are therefore dependent on the species richness in the reserve relative to the surrounding landscape, and the amount of leakage present. Although it is widely recognised that reserves need to be effectively managed in order for them to be successful in conserving biodiversity [26], here we have shown for the first time that even well managed protected area networks can provide unexpectedly low biodiversity benefits for a landscape where leakage is operating. The size of the reserves and effectiveness of management determine the biodiversity protected in the reserve. Reserves that are too small or lack appropriate management may suffer from biodiversity loss independent of leakage. In this instance, leakage will still cause species loss in areas outside of the reserve, however the ratio of species loss between the protected and unprotected will be lower

The purchase of land for conservation is often subject to opportunity. In the absence of leakage, the opportunistic purchase of any land parcel creates a positive biodiversity outcome. However, if leakage is operating in the landscape, it is important that conservation managers consider both the species richness of the parcel targeted for protection, and the species richness of the surrounding ‘at risk’ areas that will remain unprotected. If the opportunistically available parcel of land is of considerably lower species richness than the surrounding landscape, the protection of this land may perversely result in net negative biodiversity outcomes as threats are shifted onto more valuable parcels. Given the impacts of leakage, the historical trend of preferentially protecting “the land that no one else wanted” [27] not only incurs opportunity costs that undermine net conservation outcomes, it can generate outright costs by displacing degradation on more valuable land that results in a net decline in species richness.

Various forms of leakage can operate in a single landscape, at scales ranging from local to international. This makes it challenging to quantify the magnitude of the effect, and to identify where the threats may be displaced to. If leakage results from local “activity-shifting” (e.g., when people move subsistence extraction to just outside reserve boundaries), the leakage impacts can be estimated by measuring the change in extant species richness on unprotected land within travel distance of the reserve [28]. However, measuring the impact of leakage becomes more problematic under leakage that is driven by demand across a broader-scale market for the resources available in the landscape, and where reserves alter the supply and demand for resources. If a new protected area could displace the degrading activity at a larger spatial scale than activity-shifting leakage [14], particularly a global scale (e.g., the international timber market) estimation becomes much more difficult. Previous projects have estimated leakage rates by tracking the behaviour of the specific actors responsible for habitat loss in the aftermath of a conservation intervention [29], though this approach is time consuming and not always feasible. Alternatively, an econometric modeling approach could be employed [18]. Bio-economic modeling can help to predict the expected change in the location and scale of degrading activities. Such process-based models would allow managers to make a priori estimates of the leakage parameter.

In these analyses we only consider cases where leakage ranges from λ = 0 to λ = 1, however, there may be cases when the leakage coefficient is greater than one. For example, if the creation of protected areas displaces farming from highly productive land into more marginal areas, a greater area may be required to supply the same amount of farmed produce. In these situations the negative impacts of leakage would be even greater, highlighting the importance of incorporating its predicted impacts into conservation planning. In the same vein, our models also assumed that leakage would have a uniform expected distribution across the landscape. In contrast, the nature of the degrading activity, and specific features in the conservation landscape (e.g., elevation, productivity, distance to markets, distance from human communities) will determine where activities will be displaced to. Additionally, we assumed that the displaced threat is shared equally by all unprotected parcels however, in reality leakage is probably going to be concentrated around the protected area and on land with similar usage, for example if a forested area was protected from logging leakage is most likely to occur in another forested area. Leakage will therefore be concentrated in a smaller area causing greater biodiversity loss in these areas.

In contrast to some previous predictions [30], our results highlight the importance of incorporating leakage into conservation decision making. We clearly demonstrate that leakage has the potential to undermine conservation efforts, particularly where species richness is evenly distributed in the landscape so that new protected areas have comparable species richness to areas left unprotected, or where conservation is implemented opportunistically. Moreover, we demonstrate that using systematic conservation planning to choose the highest value parcels has heightened importance in instances where leakage is present, as a structured approach can avoid the possibility of net negative biodiversity outcomes. By preferentially working in regions with heterogeneous biodiversity, and by foregoing conservation opportunities that are likely to displace threats into high biodiversity locations, the negative impacts of leakage on biodiversity outcomes can be minimized. To ensure that the establishment of a protected area does not simply result in a shift of the degrading activity to areas outside the protected area the underlying drivers of biodiversity loss and the behaviour of the people who use the area need to be addressed [28]. Further research into the process and drivers of leakage needs to be undertaken to better inform decision-making for conservation acquisition.
